# Arterial stiffness and progression of cerebral white matter hyperintensities in patients with type 2 diabetes and matched controls: a 5-year cohort study

**DOI:** 10.1186/s13098-021-00691-y

**Published:** 2021-06-26

**Authors:** Kristian L. Funck, Esben Laugesen, Pernille Høyem, Brian Stausbøl-Grøn, Won Y. Kim, Leif Østergaard, Dora Grauballe, Troels K. Hansen, Christian S. Buhl, Per L. Poulsen

**Affiliations:** 1 Department of Endocrinology and Internal Medicine, Aarhus University Hospital, Palle Juul-Jensens Boulevard 99, 8200 Aarhus N Aarhus, Denmark; 2 Steno Diabetes Center Aarhus, Aarhus University Hospital, Aarhus, Denmark; 3 Department of Clinical Medicine, Aarhus University, Aarhus, Denmark; 4 The Danish Diabetes Academy, Odense, Denmark; 5 MR Research Centre, Department of Clinical Medicine, Aarhus University, Aarhus, Denmark; 6 Department of Cardiology, Aarhus University Hospital, Aarhus, Denmark; 7 Center of Functionally Integrative Neuroscience, Aarhus University, Aarhus, Denmark; 8 Neuroradiology Research Unit, Section of Neuroradiology, Department of Radiology, Aarhus University Hospital, Aarhus, Denmark

**Keywords:** Vascular stiffness, Carotid-femoral pulse wave velocity, White matter hyperintensities, Cerebral small vessel disease, Type 2 diabetes

## Abstract

**Background:**

Stroke is a serious complication in patients with type 2 diabetes (T2DM). Arterial stiffness may improve stroke prediction. We investigated the association between carotid-femoral pulse wave velocity [PWV] and the progression of cerebral white matter hyperintensities (WMH), a marker of stroke risk, in patients with T2DM and controls.

**Methods:**

In a 5-year cohort study, data from 45 patients and 59 non-diabetic controls were available for analysis. At baseline, participants had a mean (± SD) age of 59  ±  10 years and patients had a median (range) diabetes duration of 1.8 (0.8–3.2) years. PWV was obtained by tonometry and WMH volume by an automated segmentation algorithm based on cerebral T2-FLAIR and T1 MRI (corrected by intracranial volume, cWMH). High PWV was defined above 8.94 m/s (corresponding to the reference of high PWV above 10 m/s using the standardized path length method).

**Results:**

Patients with T2DM had a higher PWV than controls (8.8  ±  2.2 vs. 7.9  ±  1.4 m/s, p  <  0.01). WMH progression were similar in the two groups (p  =  0.5). One m/s increase in baseline PWV was associated with a 16% [95% CI 1–32%], p  <  0.05) increase in cWMH volume at 5 years follow-up after adjustment for age, sex, diabetes, pulse pressure and smoking. High PWV was associated with cWMH progression in the combined cohort (p  <  0.05). We found no interaction between diabetes and PWV on cWMH progression.

**Conclusions:**

PWV is associated with cWMH progression in patients with type 2 diabetes and non-diabetic controls. Our results indicate that arterial stiffness may be involved early in the pathophysiology leading to cerebrovascular diseases.

**Supplementary Information:**

The online version contains supplementary material available at 10.1186/s13098-021-00691-y.

## Introduction

Patients with type 2 diabetes are at high risk of cerebrovascular complications including stroke, vascular dementia and cognitive impairment [1, 2]. The epidemic burden of type 2 diabetes worldwide highlights the need for new biomarkers to improve individual risk prediction and to elucidate mechanisms underlying cerebrovascular disease.

Carotid-femoral pulse wave velocity (PWV), a simple and non-invasive measure of arterial stiffness, is a strong predictor of stroke [3, 4]. Stiffening of the elastic arteries may impair the normal protective impedance mismatch between elastic and muscular arteries permitting excess pulsatile energy to be transmitted into the microcirculation [5]. High-flow low-impedance organs, such as the brain, are particularly vulnerable to these effects, and the pulsatile energy may inflict target organ damage [6]. This notion is supported by cross-sectional studies demonstrating associations between PWV and cerebral white matter hyperintensities (WMH) in various populations [7], including patients with type 2 diabetes [8]. WMHs are established markers of future cerebrovascular disease [9, 10]. It is currently unknown whether PWV is associated with WMH progression and whether this association differs between patients with and without type 2 diabetes.

The aim of this cohort study was to investigate the association between PWV and WMH progression during 5 years follow-up in patients with type 2 diabetes without a history of symptomatic cerebrovascular disease and in healthy sex- and age-matched controls.

## Methods

### Design and subjects

We performed a 5-year follow-up study comprising 100 patients with type 2 diabetes and 100 age- (± 2 years) and sex-matched controls at the Department of Endocrinology and Internal Medicine, Aarhus University Hospital, Denmark. Other data regarding this population have previously been published [8]. Inclusion criteria at baseline were age  >  18 years and, for patients,  <  5 years since diagnosis of diabetes. Controls were excluded if diabetes was diagnosed by fasting glucose and oral glucose tolerance tests. Other exclusion criteria were: acute or chronic infectious disease, end-stage renal failure, pregnancy or lactation, prior or current cancer and contraindications to magnetic resonance imaging (MRI) (claustrophobia, magnetic material in the body and a body weight  >  120 kg). Participants were invited for a baseline visit (2009–2011) and a 5-year follow-up visit (2014–2016). We obtained blood and urine samples and assessed arterial stiffness, office and ambulatory blood pressure (BP), and anthropometrics at both visits. Moreover, we performed a cerebral MRI at both visits in order to assess WMH burden.

### Pulse wave velocity measurements

Examinations were conducted from 8 to 12 a.m. The study subjects had abstained from smoking and intake of food or caffeinated beverages for at least 2 h before examination. Measurements of PWV were performed using an applanation tonometer (SPT-301B; Millar, Houston, TX, USA) and SphygmoCor equipment and software, version 8.0 (AtCor Medical, Sydney, Australia). After a minimum of 5 min of rest in the supine position, sequential electrocardiogram-referenced tonometry-based recordings of the pulse wave at the carotid and the femoral artery were performed to determine the PWV. The transit time was determined by the intersecting tangent algorithm method [11], and the path length was calculated by subtracting the distance between the site of the carotid artery pulse measurement and the suprasternal notch from the distance between the site of the femoral artery pulse measurement and the suprasternal notch, all measured directly using a tape measure. The direct path length method was not applied in our primary analysis, as the study was initiated prior to publication of the consensus document on path length measurements. A PWV of 8.94 m/s in our data set corresponds to 10 m/s when converting PWV to the standard path length [4]; a cut-off value that is associated with greater risk of stroke and cardiovascular events [12]. The mean of two PWV measurements per examination was calculated. PWV assessed by the SphygmoCor device is characterized by good reproducibility in patients with type 2 diabetes and healthy individuals [13].

### Magnetic resonance imaging

At baseline, a cerebral MRI was performed with an eight-channel SENSE head coil on a 1.5 T MRI scanner (Achieva, Philips, Best, Netherlands) to obtain both axial T2-FLAIR scans (256 × 256 × 22 with acquisition matrix of 256 × 191; slice thickness 5 mm; TE  =  130 ms; TR  =  6000 ms; TI  =  2200 ms; flip angle 90º) and T1-weighted 3D FFE scans (256 × 256 × 150 with acquisition matrix of 256 × 256; slice thickness 2 mm; TE  =  4.60 ms; TR  =  25 ms; flip angle 30º).

At follow-up, cerebral MRI was performed using a 32-channel head coil on a 3 T scanner (MAGNETOM Skyra system, Siemens Healthcare, Erlangen, Germany) to obtain both a T2-FLAIR sequence (320 × 310 × 45 with acquisition matrix of 320 × 217; slice thickness 3 mm; TE  =  117 ms; TR  =  9000 ms; TI  =  2500 ms; flip angle 150º), and T1-weighted uniform MP2RAGE sequence (256 × 240 × 176 with acquisition matrix of 256 × 240; slice thickness 1 mm; TE  =  2.98 ms; TR  =  5000 ms; flip angle 0º).

After manual inspection of image quality, the obtained sequences were processed with an  × 86-based workstation. The T1W-images from follow-up (MP2RAGE) were prepared for analysis with removal of background noise using the robust T1W method. We used the same β-value for all image sequences which yielded an adequate noise suppression without introduction of significant intensity bias [14].

The volume and number of WMH at baseline and follow-up was quantified by an automated segmentation of both T1 and T2-FLAIR sequences using the Lesion Growth Algorithm (LGA) [15] as implemented in the LST toolbox 1.2.3 (www.statistical-modelling.de/lst.html) working in Statistical Parametric Mapping (SPM) version 8 (http://www.fil.ion.ucl.ac.uk/spm/software/spm8/) as described elsewhere[16]. In the analysis we used an initial threshold (κ  =  0.3) which was confirmed by visual inspection. As patients were evaluated with different scanners at baseline and follow-up, we conducted secondary analysis with the LGA (T1 and T2FLAIR images, κ  =  0.3) and the Lesion Prediction Algorithm (LPA) (T2 FLAIR images only) [17] using SPM12 and LST toolbox 3.0.0 in order to validate the primary analysis (LGA SPM8 has shown better correlation with visual volume assessment[16] and were thus used in our primary analysis). WMH volumes were divided by intracranial volume in order to correct for individual differences in head size (cWMH). Furthermore, we included another secondary analysis of white matter hypointensity volumes using volumetric segmentation of the recorded T1-weighted images (Freesurfer 5.3, https://surfer.nmr.mgh.harvard.edu) [18]. Longitudinal analysis was performed according to Reuter et al. [19].

Cerebral infarctions were defined as areas with volume loss surrounded by signals consistent with gliosis and were classified as lacunar when their size was less than 15 mm.

### Other measurements

Ambulatory BP was measured at 20-min intervals for 24 h using Spacelab 90,217 (Spacelabs Healthcare, Issaquah, WA, USA). Office BP was measured on the right arm with an appropriately sized cuff, and mean systolic and diastolic BPs were calculated as the average of three measurements obtained after a minimum of 5 min of rest in seated position (Riester Champion N, Riester GmbH, Jungingen, Germany). Urinary albumin excretion was evaluated by albumin-to-creatinine ratios in three morning urine samples. Finally, the participants’ medical histories were obtained by questionnaire.

### Statistics

Variables with a normal distribution are presented as either mean  ±  SD (participant characteristics) or mean and 95% CI (analysis of association), and skewed data are presented as median (interquartile range). Dichotomous data are presented as n (%).

The distributions of continuous variables were tested with histograms and QQ-plots.

If normally distributed, means of two groups were compared with Student’s paired t test for matched data or with Student’s unpaired t test for independent data. Skewed data were log-transformed before using a t test. If normal distribution was not achieved by log transformation, the Wilcoxon signed rank test or the Wilcoxon–Mann–Whitney test was applied as appropriate. Dichotomous variables were compared with McNemar’s test or the Chi^2^ test as appropriate.

We used linear and logistic regression to evaluate the association between PWV at baseline and cWMH progression. Robust standard errors were calculated to account for clustering in repeated measurements. The following covariates were considered for inclusion in multivariate logistic regression models: age, sex, diabetes (yes/no), office pulse pressure (PP)[20] and smoking (no smoking vs. current/former smoking). Additionally, we exchanged PWV as a continuous variable with PWV dichotomized  < / >  8.94 m/s (corresponding to the clinical cut-off of 10 m/s when using the standard path length). There were small differences in follow-up time between patients with type 2 diabetes and controls, and therefore we repeated the analysis with cWMH volume progression corrected for follow-up time. Furthermore, as lacunar infarcts may have been included in the automatic cWMH segmentation, we repeated the analyses without patients with presence of infarcts. Finally, we tested the interaction between diabetes status and PWV on the effect on cWMH progression.

Two-sided P values  <  0.05 were considered statistically significant. Statistical analyses were performed with Stata software version 13 (StataCorp, College Station, TX, USA).

## Results

### Participant characteristics

A total of 63 patients with type 2 diabetes and 72 controls attended the follow-up visit. Data from participants with a history of stroke or transient ischaemic attack at baseline (n  =  4), participants with missing PWV (n  =  10) or missing MRI (n  =  17) were excluded from the analysis (Additional file 1: Figure S1). Data from 45 patients and 59 controls were available for our final analysis (Baseline characteristics of participants attending versus those not attending the follow-up visit are listed in Additional file 1: Table S1).

Patients with type 2 diabetes had, compared with controls, a higher BMI, heart rate and PWV, but more favourable plasma lipids, and BP profiles at baseline (Table 1). The good risk factor control observed in the diabetes group could probably be ascribed to the fact that a high proportion of the patients with diabetes received antihypertensive drugs and statins.

**Table 1:** Participant characteristics at baseline

Participant characteristics	DM (n = 45)	Controls (n = 59)	p value
Male n (%)	22 (49)	28 (47)	0.89
Age (years)	59.3 ± 9.8	57.9 ± 9.8	0.47
Diabetes duration (years)	1.8 (0.8–3.2)	Na	–
Follow-up (years)	5.6 ± 0.4	5.4 ± 0.3	< 0.05
BMI (kg/m^2^)	29.3 ± 5.1	25.9 ± 3.3	0.08
HbA1c (mmol/mol)	47 ± 6	38 ± 4	< 0.05
HbA1c (%)	6.5 ± 0.6	5.6 ± 0.4	< 0.05
Total cholesterol (mmol/l)	4.4 ± 0.8	5.7 ± 1.0	< 0.05
LDL (mmol/l)	2.2 ± 0.7	3.4 ± 1.0	< 0.05
Office systolic blood pressure (mmHg)	126 ± 9	131 ± 14	0.07
Office diastolic blood pressure (mmHg)	79 ± 7	83 ± 9	< 0.05
Office heart rate (bpm)	66 ± 9	62 ± 10	< 0.05
24-h systolic blood pressure (mmHg)	125 ± 9	124 ± 11	0.58
24-h diastolic blood pressure (mmHg)	74 ± 6	75 ± 7	0.47
24-h heart rate (bpm)	73 ± 10	68 ± 9	< 0.05
Urine albumin creatinine ratio (mg/mmol)^a^	0.4 (0.3–1.0)	0.2 (0.2–0.4)	< 0.05
Antidiabetic medicine (oral and GLP-1-analogues) n (%)	32 (71)	0 (0)	< 0.05
Insulin n (%)	5 (11)	0 (0)	< 0.05
Antihypertensives n (%)	28 (62)	15 (25)	< 0.05
Statin n (%)	33 (73)	9 (15)	< 0.05
Aspirin n (%)	25 (56)	2 (3)	< 0.05
Current or former smoker n (%)	24 (53)	29 (49)	0.67
PWV (m/s)	8.8 ± 2.1	7.9 ± 1.4	< 0.05

Parametric data presented as mean  ±  SD

*PWV *carotid-femoral pulse wave velocity

^a^Median (interquartile range)

### White matter hyperintensity progression

Patients with type 2 diabetes had comparable WMH volumes compared to controls both at baseline and follow-up. In accordance, progression in WMH volumes was similar in the two groups during the study period (Table 2) and the median volume increase in WMH was 984mm^3^ for the combined cohort. Similarly, the number of WMHs was low at baseline but increased similarly in both groups. Using cWMH did not change the results (p  <  0.44). At baseline, three (7%) patients and three (8%) controls had signs of subclinical cerebral infarctions on MRI (p  =  1.0), which did not change at follow-up. No participants had signs of brain tumors.

**Table 2:** Cerebral white matter lesions

	Type 2 diabetes (n = 45)	Controls (n = 59)	p value
Volume of white matter lesions (mm^3^)			
Baseline	258 (52–588)	196 (35–825)	0.82
Follow-up	1402 (452–2816)	1250 (388–3013)	0.60
Progression	1003 (402–2304)	964 (288–2252)	0.50
Number of white matter lesions (n)			
Baseline	2 (1–7)	3 (1–7)	0.92
Follow-up	12 (7–16)	9 (4–15)	0.35
Progression	7 (4–11)	6 (3–9)	0.24

Median (interquartile range)

### PWV and cWMH progression

**Fig. 1:**
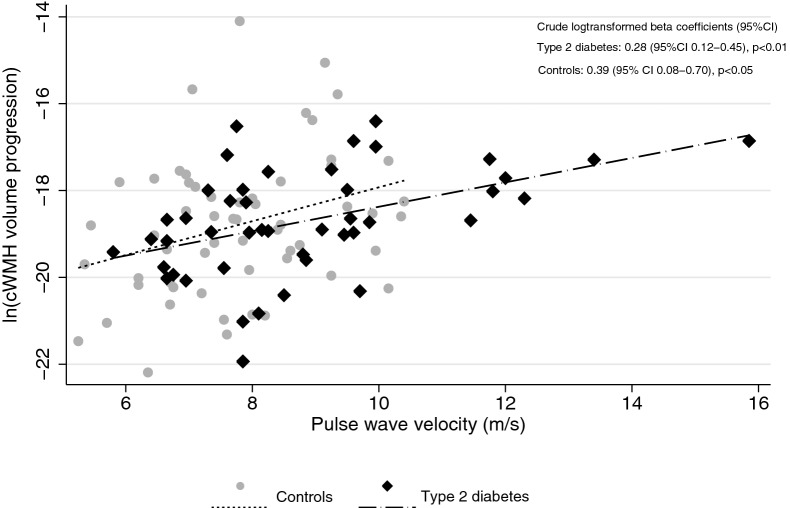
Pulse wave velocity and 5-year white matter hyperintensitiy volume progression. Diamond and dashed line  =  Type 2 diabetes. Circles and solid line  =  Controls. *cWMH* white matter hyperintensity volume progression corrected for intracranial volume

Baseline PWV was associated with increased cWMH volume progression in both crude and adjusted linear regression analysis in the combined cohort (Fig. 1; Table 3). In adjusted separate group analysis, this association was attenuated.

**Table 3:** Pulse wave velocity and white matter hyperintensity progression

Linear regression						
	All (n = 104)	p	DM (n = 45)	p	Controls (n = 59)	p
% change in cWMH volume per 1 m/s increase in PWV (95% CI)s						
Crude	34 (20–51)	< 0.01	32 (12–56)	< 0.01	48 (8–102)	< 0.05
Adjusted^a^	16 (1–32)	< 0.05	18 (− 5 to 47)	0.13	31 (− 8 to 87)	0.13
% change cWMH volume if PWV > 8.94 m/s						
Crude	208 (73–451)	< 0.01	214 (57–526)	< 0.01	218 (18–756)	< 0.05
Adjusted^a^	92 (2–263)	< 0.05	88 (− 20 to 339)	0.14	161 (− 5 to 618)	0.06

*cWMH* white matter hyperintensities corrected for intracranial volume; *PWV* pulse wave velocity

^a^Adjusted model: age, sex, diabetes status (yes/no), baseline pulse pressure and smoking status (no smoking or current/former smoking). In separate group analysis, diabetes status was omitted

**Fig. 2:**
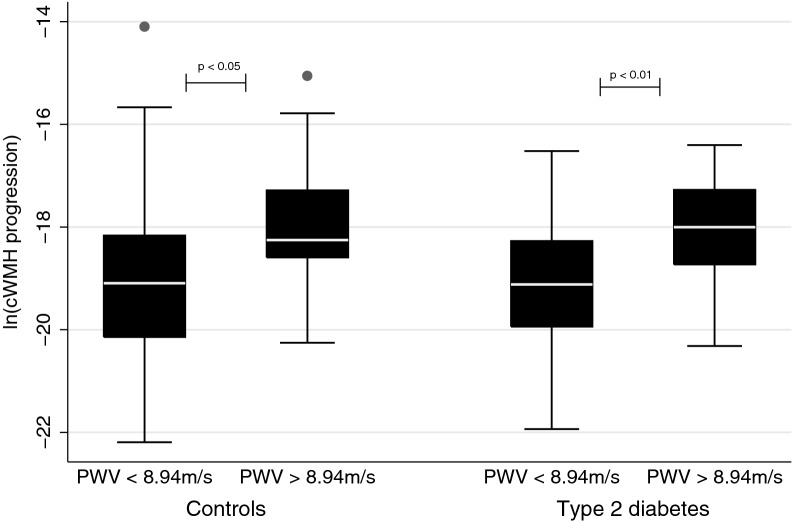
Low vs. high pulse wave velocity and white matter hyperintensity volume progression. White center line  =  median, box  =  interquartile range, whiskers: range. *cWMH *white matter hyperintensities corrected for intracranial volume. *PWV *pulse wave velocity

PWV  >  8.94 m/s (corresponding to a high PWV above 10 m/s using the standard path length measurement), was associated with high cWMH volume progression in the combined cohort and in the diabetes and control groups separately (Fig. 2). The association remained significant in the combined cohort in multivariate analysis (Table 3). The results were similar in analysis with cWMH volume progression corrected for follow-up time and in analysis exchanging PP with mean arterial pressure (data not shown). Furthermore, the results were not attenuated when we added antidiabetic, anthihypertensive and/or lipid-lowering drug use (both separately or all three variables together) nor when we added hba1c to the multivariate regression model of the combined cohort. Finally, we rerun the analysis without the participants with presence of lacunar infarcts, with similar results in crude/adjusted analysis of continuous PWV (p  <  0.05), however attenuated in adjusted analysis with dichotomized PWV (p  =  0.08). Diabetes did not modify the association between PWV and cWMH volume progression (interaction term  − 11%, 95%CI  − 32 to 18%, p  =  0.44).

In secondary analyses, we used the LST toolbox 3.0.0 and SPM12 to perform lesion segmentation of both T2 FLAIR images only (LPA12) and of a combination of T1 and T2 FLAIR (LGA12). White matter hypointensity volumes were also estimated using Freesurfer 5.3 based volumetric segmentation of T1-weighted images (as a proxy for T2 FLAIR WMH). We found results with similar direction of association although the associations were attenuated in the analysis of WMH segmentation based on T1-weighted images (Additional file 1: Table S2).

## Discussion

To our knowledge, our study is the first to evaluate PWV and cWMH volume progression in a cohort of patients with type 2 diabetes with no clinical history of cerebrovascular disease and age- and sex-matched controls. We found that PWV was associated with higher cWMH volume progression in the combined cohort, independent of known risk factors such as age, sex, blood pressure, and smoking status.

Cerebral WMHs are established surrogate markers of cerebrovascular disease like stroke, dementia and cognitive decline [9, 10]. Cerebral small-vessel damage is considered the causative factor in WMH development [21–23], and major determinants of WMH include age, hypertension, smoking, and diabetes [24–26]. We found no difference in WMH burden or progression between patients with type 2 diabetes and controls at either baseline [8] or follow-up. However, the potential differences between the two groups may be ameliorated by the good glycaemic, lipid and BP control observed in the patient group. Additionally, as patients were enrolled shortly after their diagnosis of diabetes, it may be speculated that WMH progression would start to progress faster at later stages of the disease (i.e., after the 5-year follow-up visit). This is supported by the findings of Debette et al. [27] showing no association between midlife diabetes and WMH progression. In contrast, the study results of King et al. [28] showed WMH progression at a faster pace in patients with type 2 diabetes compared to healthy individuals after the age of 50 years, but not before (the mean age at inclusion in our study was 58.5 years). Yet another explanation may be that diabetes status modifies the effect of WMH on stroke risk, e.g., the presence of WMH in diabetes patients may confer a higher risk of stroke compared to similar WMH presence in non-diabetic individuals. Prospective studies are needed to further investigate these questions.

Arterial stiffness may be a key factor in the pathogenesis of WMH. PWV has been linked to WMH in several cross-sectional studies [7], and we have previously reported a cross-sectional association between PWV and WMH in newly diagnosed patients with type 2 diabetes [8]. In contrast, Nomura and colleagues found no independent association between brachial-ankle PWV and the presence of silent infarctions in older Japanese patients with type 2 diabetes [29]. Two studies have observed longitudinal associations between baseline PWV and WMH [30, 31]. Rosano et al. [30] found baseline PWV to be associated with WMH 10 years later in a population study of elderly subjects (12% with diabetes). Tsao et al. [31] did not find an association between baseline PWV and WMH progression in a population-based cohort in which 9% were patients with diabetes. However, no previous studies prospectively investigated the association between PWV and progression of WMH in a diabetes population.

Interestingly, we found an association between PWV and cWMH progression in the combined cohort and our test for interaction did not suggest diabetes to modify the association between PWV and WMH progression. The discrepancy between our results and that of Tsao, might be due to different methodologies. Alternatively, the higher proportion of diabetes patients in our study may change the distribution of PWV towards higher values. In line with this speculation, it has been suggested that PWV only becomes a risk factor when it exceeds a certain threshold [32], e.g., 10 m/s which is the suggested cut-off value for high arterial stiffness [4]. Another important question is, whether high PWV affects the brain globally or preferentially in specific regions of the brain. In the latter case, the global assessment of WMH burden may have attenuated the observed associations between PWV and WMH progression. In general, stiffness of the elastic arteries impairs the normal impedance mismatch between elastic and muscular arteries, leading to a transfer of high pulsatile energy to the microcirculation [5]. This pulsatile barotrauma as well as the compensatory remodelling of the arteries that leads to a reduced vasodilatatory reserve, may affect the brain globally [5]. However, it has also been suggested that increased pulse wave velocity and pressure pulsatility may lead to pressure changes of the cerebrovascular fluid. In turn this may affect the periventricular parenchyma and cause periventricular WMLs [33]. Future studies evaluating WMH progression in specific brain regions may help answer these questions.

The present study has some limitations. Firstly, a 1.5 T MR scanner was used at baseline and a 3.0 T MR scanner at follow-up. This may cause a higher volume estimation at follow-up, partly because of better detection of punctate lesions [34, 35]. Theoretically, our results may therefore reflect the cross-sectional association between PWV and WMH as reported previously [8]. However, in both LGA8 analysis and sensitivity analysis using different segmentation techniques (see “Methods”), we have visually observed that WMH progression vary considerably at similar baseline WMH volumes which suggests that our results reflect actual WMH progression. Furthermore, all of the analyses showed the same direction of association as the main analysis based on LGA8. Finally, the scanner changed in all patients at follow-up, independent of PWV value, and therefore it might not affect the association of PWV with cWMH. Secondly, due to the modest sample size, analysis was at risk of type 2 error, and multivariable regression analysis was restricted to only few confounding covariates. Thirdly, the dropout of 37 patients and 28 controls before the follow-up visit might affect the external validity of the study. However, importantly, the baseline characteristics between participants who participated in the follow-up visit were not different from the participants who did not attend (Additional file 1: Table S1). Thus, the findings of this study may be applicable to other patients newly diagnosed with diabetes and individuals without diabetes.

## Conclusions

Arterial stiffness is associated with cWMH progression in patients with type 2 diabetes and matched controls. Arterial stiffness may candidate as a new risk marker for future cerebrovascular events and could potentially be an important target for intervention.

## Supplementary Information

**Additional file 1: Figure S1.** Participant flow. **Table S1.** Baseline characteristics in participants attending versus not attending the follow-up visit. **Table S2.** Pulse wave velocity and white matter hyperintensity progression.

## Data Availability

The data of this study is available on request.

## Abbreviations

BMI: Body mass index
BP: Blood pressure
LGA: Lesion growth algorithm
LPA: Lesion prediction algorithm
MRI: Magnetic resonance imaging
PWV: Pulse wave velocity
WMH: White matter hyperintensities
