# Supplementary material for: Arterial stiffness and progression of cerebral white matter hyperintensities in patients with type 2 diabetes and matched controls: a 5-year cohort study
Source: Diabetol Metab Syndr. 2021 Jun 26;13:71. doi: 10.1186/s13098-021-00691-y (PMC8236189; doi:10.1186/s13098-021-00691-y)
Supplement: Supplementary file 1 — Additional file 1: Figure S1. Participant flow. Table S1. Baseline characteristics in participants attending versus not attending the follow-up visit. Table S2. Pulse wave velocity and white matter hyperintensity progression. [file 13098_2021_691_MOESM1_ESM.pptx]

## Slide 1
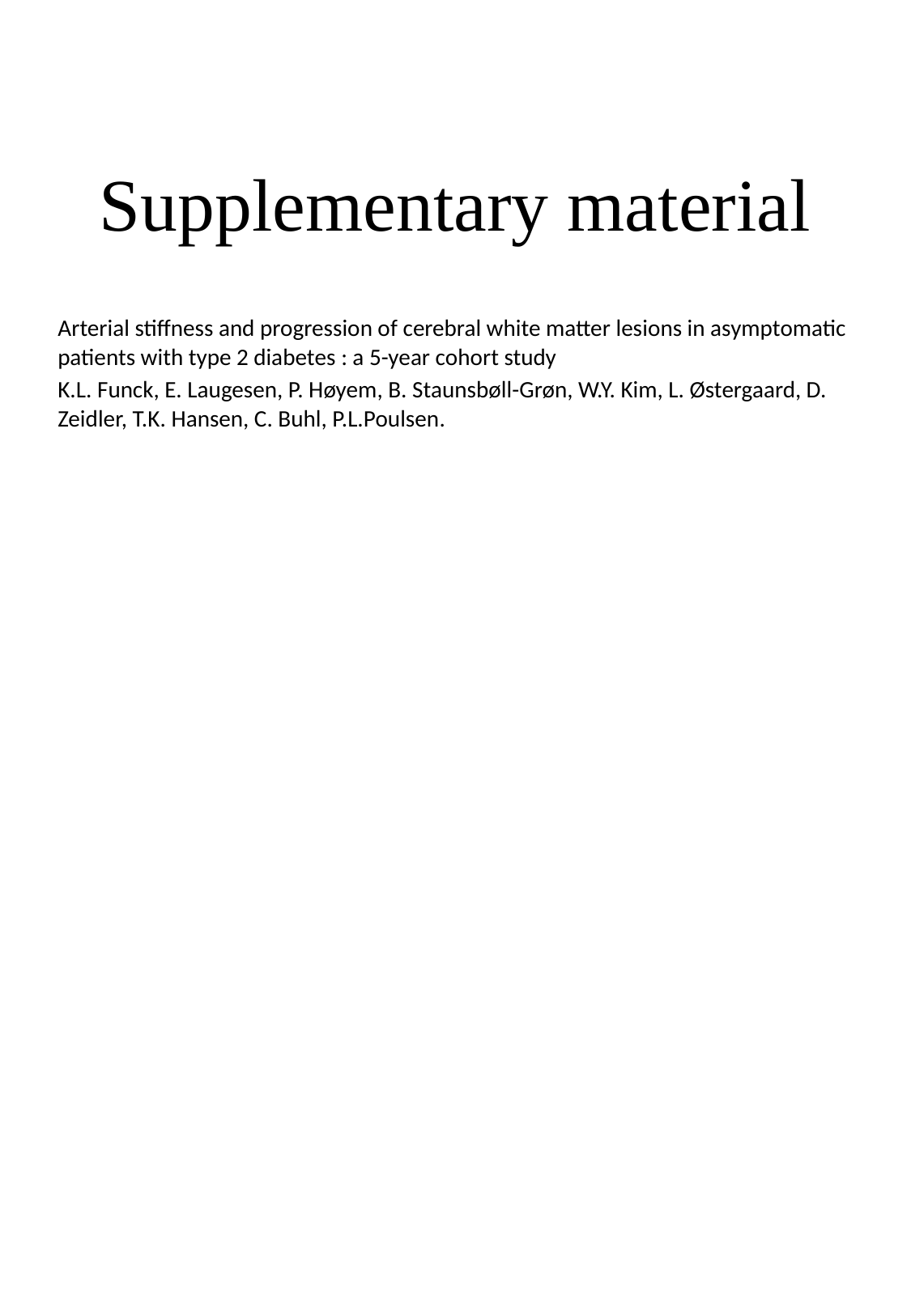

Supplementary material
Arterial stiffness and progression of cerebral white matter lesions in asymptomatic patients with type 2 diabetes : a 5-year cohort study
K.L. Funck, E. Laugesen, P. Høyem, B. Staunsbøll-Grøn, W.Y. Kim, L. Østergaard, D. Zeidler, T.K. Hansen, C. Buhl, P.L.Poulsen.

## Slide 2
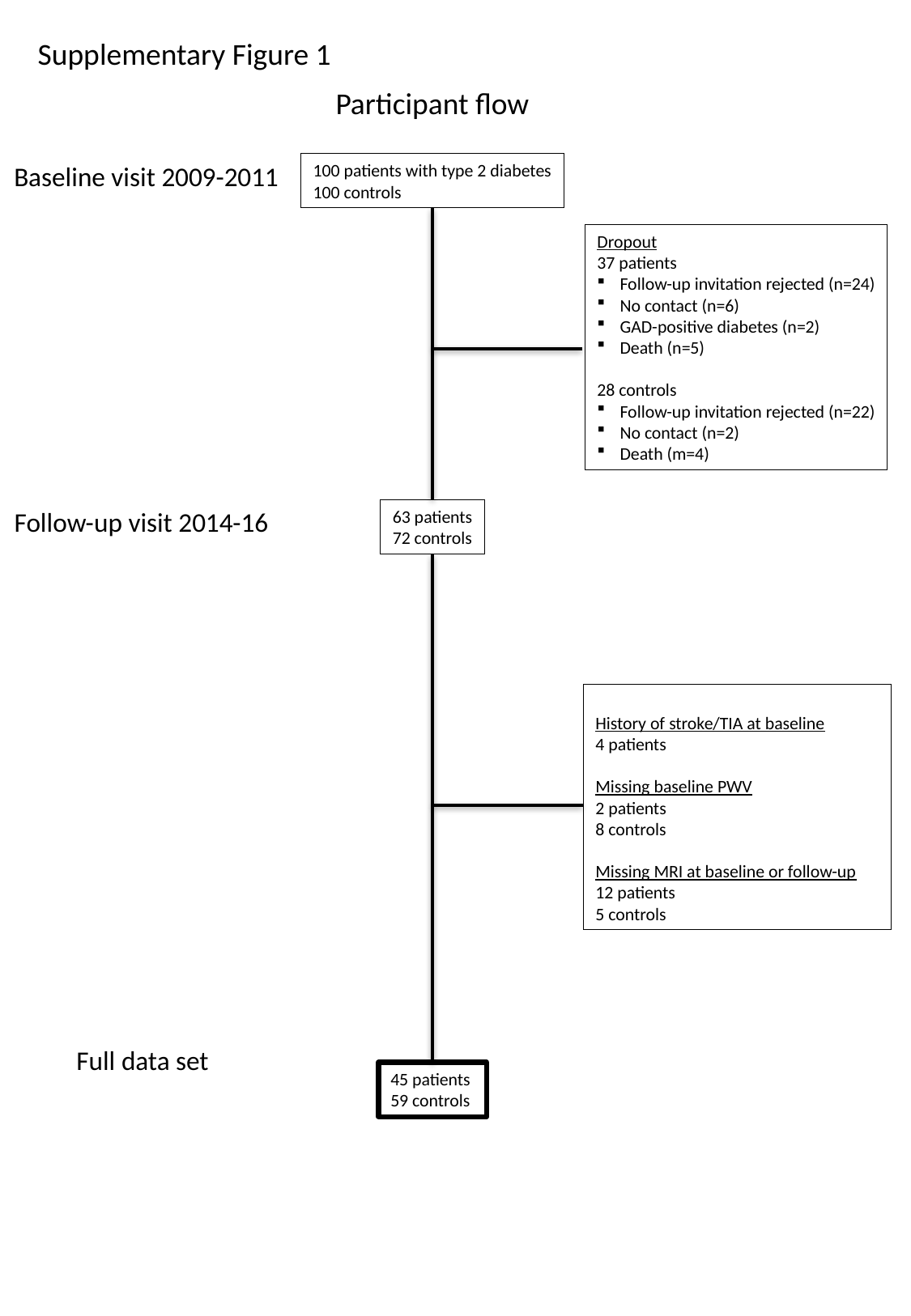

Supplementary Figure 1
Participant flow
Baseline visit 2009-2011
100 patients with type 2 diabetes
100 controls
Dropout
37 patients
Follow-up invitation rejected (n=24)
No contact (n=6)
GAD-positive diabetes (n=2)
Death (n=5)
28 controls
Follow-up invitation rejected (n=22)
No contact (n=2)
Death (m=4)
Follow-up visit 2014-16
63 patients
72 controls
History of stroke/TIA at baseline
4 patients
Missing baseline PWV
2 patients
8 controls
Missing MRI at baseline or follow-up
12 patients
5 controls
Full data set
45 patients
59 controls

## Slide 3
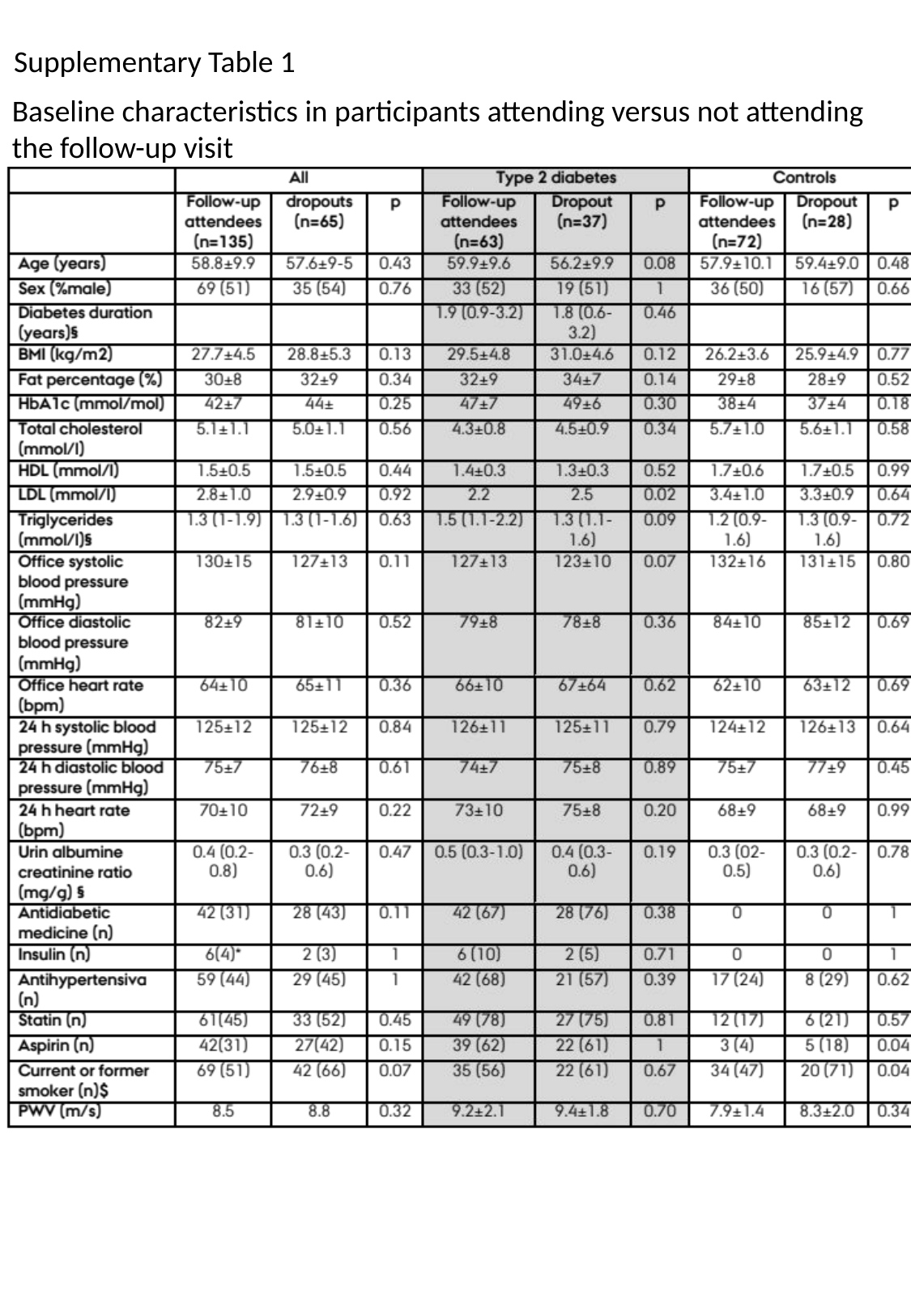

Supplementary Table 1
Baseline characteristics in participants attending versus not attending the follow-up visit

## Slide 4
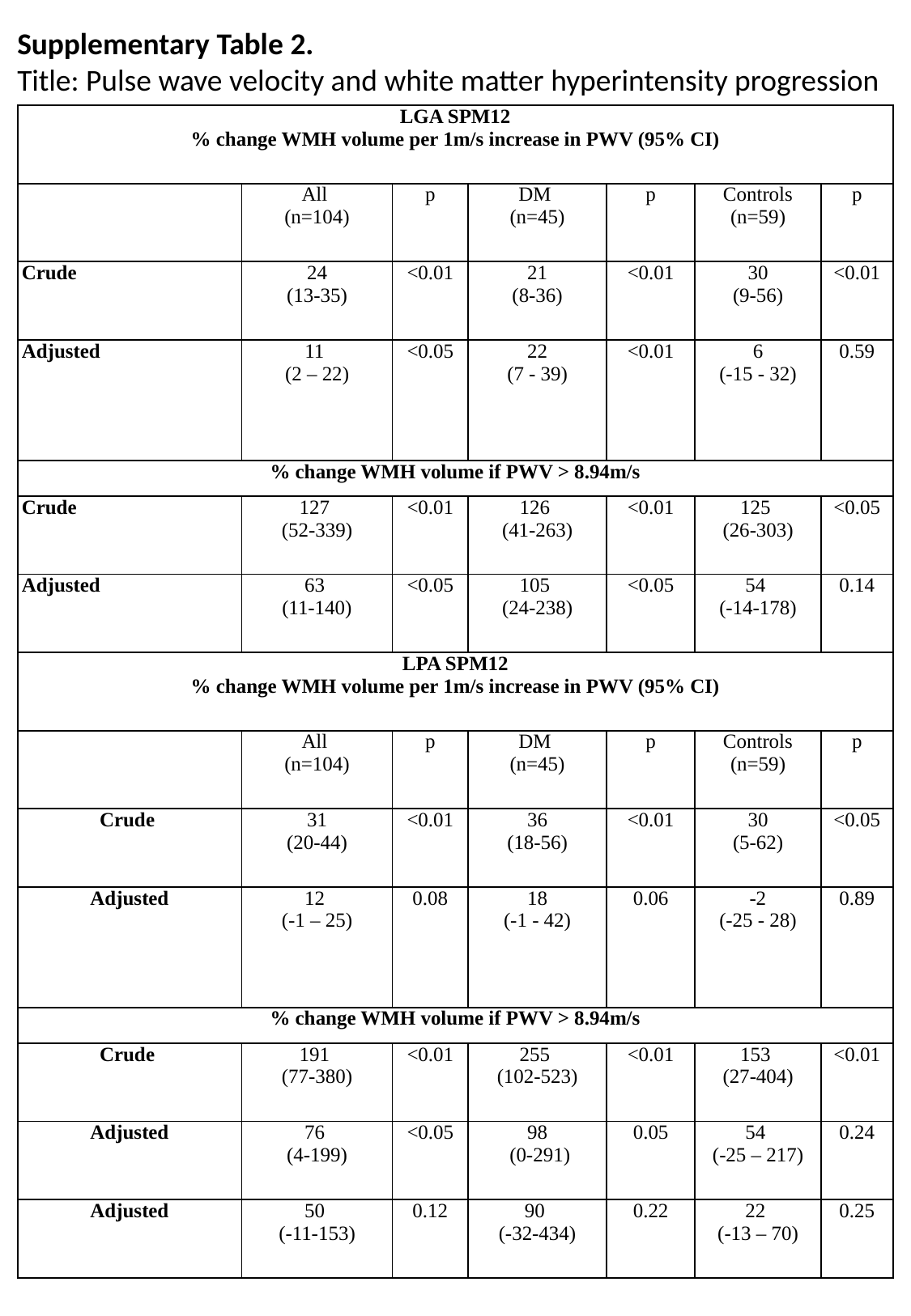

Supplementary Table 2.
Title: Pulse wave velocity and white matter hyperintensity progression
| LGA SPM12 % change WMH volume per 1m/s increase in PWV (95% CI) | | | | | | |
| --- | --- | --- | --- | --- | --- | --- |
| | All (n=104) | p | DM (n=45) | p | Controls (n=59) | p |
| Crude | 24 (13-35) | <0.01 | 21 (8-36) | <0.01 | 30 (9-56) | <0.01 |
| Adjusted | 11 (2 – 22) | <0.05 | 22 (7 - 39) | <0.01 | 6 (-15 - 32) | 0.59 |
| % change WMH volume if PWV > 8.94m/s | | | | | | |
| Crude | 127 (52-339) | <0.01 | 126 (41-263) | <0.01 | 125 (26-303) | <0.05 |
| Adjusted | 63 (11-140) | <0.05 | 105 (24-238) | <0.05 | 54 (-14-178) | 0.14 |
| LPA SPM12 % change WMH volume per 1m/s increase in PWV (95% CI) | | | | | | |
| | All (n=104) | p | DM (n=45) | p | Controls (n=59) | p |
| Crude | 31 (20-44) | <0.01 | 36 (18-56) | <0.01 | 30 (5-62) | <0.05 |
| Adjusted | 12 (-1 – 25) | 0.08 | 18 (-1 - 42) | 0.06 | -2 (-25 - 28) | 0.89 |
| % change WMH volume if PWV > 8.94m/s | | | | | | |
| Crude | 191 (77-380) | <0.01 | 255 (102-523) | <0.01 | 153 (27-404) | <0.01 |
| Adjusted | 76 (4-199) | <0.05 | 98 (0-291) | 0.05 | 54 (-25 – 217) | 0.24 |
| Adjusted | 50 (-11-153) | 0.12 | 90 (-32-434) | 0.22 | 22 (-13 – 70) | 0.25 |

## Slide 5
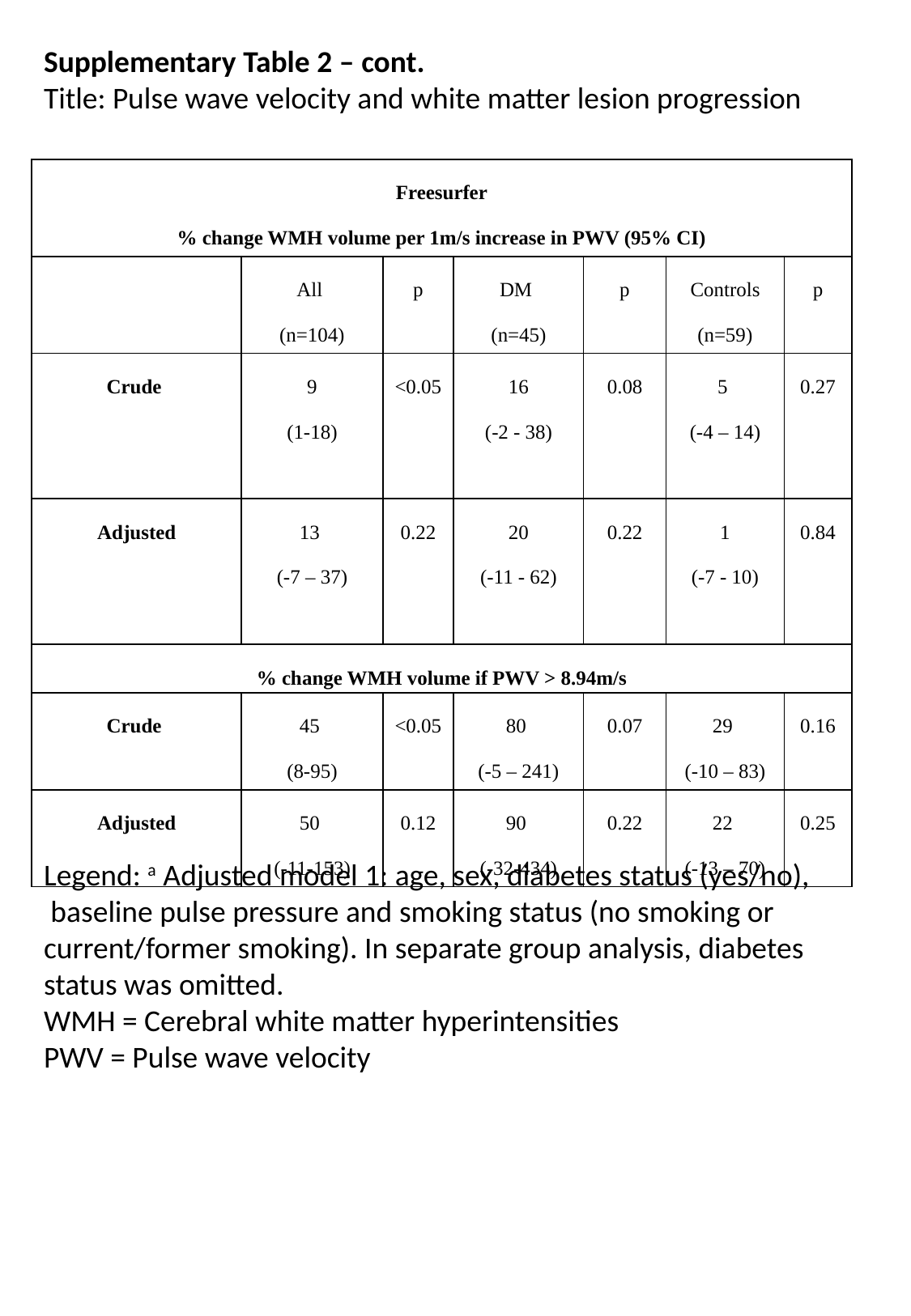

Supplementary Table 2 – cont.
Title: Pulse wave velocity and white matter lesion progression
| Freesurfer % change WMH volume per 1m/s increase in PWV (95% CI) | | | | | | |
| --- | --- | --- | --- | --- | --- | --- |
| | All (n=104) | p | DM (n=45) | p | Controls (n=59) | p |
| Crude | 9 (1-18) | <0.05 | 16 (-2 - 38) | 0.08 | 5 (-4 – 14) | 0.27 |
| Adjusted | 13 (-7 – 37) | 0.22 | 20 (-11 - 62) | 0.22 | 1 (-7 - 10) | 0.84 |
| % change WMH volume if PWV > 8.94m/s | | | | | | |
| Crude | 45 (8-95) | <0.05 | 80 (-5 – 241) | 0.07 | 29 (-10 – 83) | 0.16 |
| Adjusted | 50 (-11-153) | 0.12 | 90 (-32-434) | 0.22 | 22 (-13 – 70) | 0.25 |
Legend: a Adjusted model 1: age, sex, diabetes status (yes/no),
 baseline pulse pressure and smoking status (no smoking or current/former smoking). In separate group analysis, diabetes status was omitted.
WMH = Cerebral white matter hyperintensities
PWV = Pulse wave velocity
